# Characteristics of Traumatic Versus Atraumatic Rotator Cuff Tears in Patients Under 50 Years of Age

**DOI:** 10.7759/cureus.66450

**Published:** 2024-08-08

**Authors:** MaKenzie Chambers, Haley Tornberg, Michael Curry, Aditya Joshi, Manisha Koneru, Nicholas Pohl, Matthew T Kleiner, Catherine Fedorka

**Affiliations:** 1 Orthopaedics, Cooper Medical School of Rowan University, Camden, USA; 2 Neurointerventional Surgery, Cooper Medical School of Rowan University, Camden, USA; 3 Orthopaedic Surgery, Cooper Medical School of Rowan University, Camden, USA; 4 Orthopaedics, Cooper University Hospital, Camden, USA

**Keywords:** atraumatic, traumatic, young patients, demographic trends, rotator cuff tear

## Abstract

Background: The prevalence of rotator cuff tears (RCTs) is known to be lower in younger patients compared to older patients. Recent studies in patients less than 50 years of age who sustain an RCT have focused on etiology, pathogenesis, and clinical outcomes following treatment. There are fewer studies that have focused on the demographics and clinical characteristics that may predispose this patient population to develop a tear. The purpose of this study is to evaluate the difference in risk factors for degenerative tears compared to traumatic tears in patients under 50 years of age.

Methods: This single-center retrospective study utilized an internal registry of patients who had RCT injuries identified by the International Classification of Diseases (ICD)-10 code M75.1x and confirmed by MRI between 2018 and 2023. Patients 50 years of age or younger were included and then classified into traumatic versus atraumatic RCT etiology groups. Demographics, tear characteristics, and clinical comorbidities were compared between the cohorts. Statistical analyses included a two-sided student’s t-test, Wilcoxon rank-sum test, Chi-square test, and Fisher's exact test.

Results: A total of 177 patients under 50 years of age were identified. There was a higher prevalence of traumatic tears (59.9% vs. 40.1%; p = 0.008), the majority of whom identified as male (75.5% vs. 49.3%, p<0.001) when compared to the atraumatic cohort. Full-thickness tears were more likely to be traumatic (p = 0.04) and seen in patients insured by workers’ compensation (p = 0.05). There was no significant difference in the age or preoperative comorbidities between the two groups.

Conclusions: Our study reveals a higher incidence of traumatic RCTs in a younger patient group. Sex, severity of tear, and workers’ compensation were found to differ between traumatic and atraumatic cohorts. Further research is required to understand the interplay of these factors in younger patients' tear risk.

## Introduction

Rotator cuff tears (RCTs) are a common orthopedic injury, affecting millions of people worldwide and can pose a significant financial and health burden on the patient [1-3]. If left untreated, they can result in pain, decreased range of motion, muscle degeneration, and as a result, decreased quality of life [4,5]. Recent studies in patients younger than 50 with an RCT mainly focus on clinical outcomes following repair [6-10]. There have also been additional studies investigating the etiology and pathogenesis of RCT in this young patient population [8,11]. Many of these have shown a higher prevalence of traumatic tears while others have shown a more even split of traumatic versus another etiology (such as degenerative) [8,10,12]. In some of these analyses, the authors have highlighted differences among their patients regarding demographics and tear characteristics. Some have reported no differences in baseline demographics between patients with an RCT of traumatic etiology versus degenerative while others have shown that factors such as smoking, medical comorbidities, work status, and tear characteristics do vary based on tear etiology [7,10,13,14].

While the majority of the RCTs in this age group are traumatic in nature, there is still a large portion of younger patients who experience RCT in the absence of an inciting event [15-17]. These injuries, regardless of the etiology, can predispose the patient to future orthopedic complications and disease sequelae including cuff tear arthropathy, weakness, poor function, and impaired activities of daily living (ADLs) [18-20]. Due to the lower prevalence in young patients as compared to the older patient population, there is a consensus among the current literature on the exact etiology, pathogenesis, demographics, and postoperative outcomes in young patients with an RCT.

Though the literature on this topic is growing, there is still a lack of agreement regarding the differences, or lack thereof, in demographics and clinical characteristics among young patients who sustain an RCT due to a traumatic event or as a result of degenerative changes. The purpose of this study was to compare demographic factors between patients under 50 years of age who were treated at our institution for a traumatic RCT to those treated for a degenerative tear. It was hypothesized that patients who sustained an RCT with a documented traumatic inciting event would demonstrate differences in demographic variables when compared to those patients who sustained a tear due to non-traumatic causes. The results of this study are significant as they will add to the expanding knowledge and understanding of the specific characteristics of this patient population and aid in counseling at-risk patients on potential injury prevention and postoperative expectations and outcomes.

## Materials and methods

This is a retrospective study at a single tertiary referral center. After institutional review board approval (IRB-23-057), patients under the age of 50 who experienced an RCT between 2018 and 2023 were identified through the institution’s electronic medical record system utilizing International Classification of Diseases (ICD)-10 code M75.1X and S46.0XX. A manual chart review was then performed to confirm the presence of an RCT diagnosed by board-certified orthopedic shoulder and sports surgeons and as documented by shoulder-specific magnetic resonance imaging (MRI) imaging reports. Patients who did not have a documented diagnosis of an RCT as per an MRI report, had prior shoulder surgery, or were older than 50 were excluded. Patients were then stratified through additional chart review to determine whether the RCT was of traumatic or atraumatic origin. Traumatic etiology of injury was defined as a patient presenting after an acute injury to their shoulder and no previous shoulder pain prior to the injury with Grade 0 or 1 fatty atrophy. All MRIs were reviewed by the senior author (CJF) to classify atrophy according to Goutallier [21]. An atraumatic RCT was defined as if there was no specific mechanism of injury or inciting event prior to the onset of pain as documented in the chart notes.

Patient demographics including age, sex, race, body mass index (BMI), and hand dominance were collected. Race was collected from the electronic health record and compared between the two groups for potential differences. Additional variables collected included medical comorbidities to calculate the Charlson Comorbidity Index (CCI), and patient social factors (smoking status, alcohol use, illicit drug use, insurance coverage, workers' compensation). Additionally, MRI findings were reported as either full or partial-thickness RCT as documented by the report from a board-certified radiologist.

Statistical analyses included a two-sided student’s t-test for continuous parametric data, a Wilcoxon rank-sum test for nonparametric continuous data, a Chi-square test, and Fisher's exact test where applicable for categorical data. Univariate analysis predicting the outcome of traumatic versus atraumatic RCT tear was performed to yield unadjusted odds ratios (OR), and significant predictors were inputted into a multivariate logistic regression analysis to obtain adjusted odds ratios (aOR). Descriptive parameters for the multivariable regression model, including corrected Akaike information criterion (AIC), Bayesian information criterion (BIC), whole model test, and area under the curve (AUC), were reported. Statistical significance was set at p < 0.05 for all comparisons. Analyses were conducted with JMP Pro v17.0.0 (SAS Institute Inc., Cary, NC, USA).

## Results

A total of 177 patients met the inclusion criteria. A significantly greater proportion of patients (p = 0.008) had traumatic RCTs (106/177, 59.9%, 95% CI: 52.5-66.8%) than atraumatic RCTs (71/177, 40.1%, 95% CI: 33.2-47.5%). The average age of all patients in our study was 46 years (IQR 42-48), with a median age of 45 years (IQR 42-49) for atraumatic cuff tear patients (min 32, max 50) and 46 years (IQR 41-48) for traumatic cuff tear patients (min 23, max 50) (p=0.44; Table 1). Male patients comprised a significantly greater proportion of the traumatic cohort when compared to the atraumatic cohort (75.5% vs 49.3%, p < 0.001). A larger proportion of the traumatic cohort had a full-thickness tear compared to the atraumatic cohort (82.1% vs 69%; p = 0.04). There was no significant difference between the two cohorts regarding age, race, BMI, smoking status, alcohol use, drug use, preoperative comorbidities, American Society of Anesthesiologists (ASA) class, CCI, hand dominance, or insurance coverage (Table 1).

**Table 1:** Baseline demographic comparison between atraumatic and traumatic rotator cuff tear patients BMI: body mass index; ASA: American Society of Anesthesiologists; IQR: interquartile range; HIV: human immunodeficiency virus; AIDS: acquired immunodeficiency syndrome; COPD: chronic obstructive pulmonary disease

Variable Name	All (n = 177)	Atraumatic (n = 71)	Traumatic (n=106)	p-value
Age, median (IQR)	46 (42-48)	45 (42-49)	46 (41-48)	0.44
Sex, no. (%)				
Female	62 (35%)	36 (50.7%)	26 (24.5%)	<0.001
Male	115 (65%)	35 (49.3%)	80 (75.5%)	
Race, no. (%)				
Caucasian	99 (55.9%)	36 (50.7%)	63 (59.4%)	0.07
African-American	39 (22%)	12 (16.9%)	27 (25.5%)	
Chinese	2 (1.1%)	1 (1.4%)	1 (0.9%)	
Hispanic	24 (13.6%)	14 (19.7%)	10 (9.4%)	
Other	13 (7.3%)	8 (11.3%)	5 (4.7%)	
BMI (kg/m^2^), median (IQR)	29.9 (26.3-34.5)	29.4 (25.8-34.1)	30 (27-35.2)	0.58
Smoking Status, no. (%)				
None	98 (55.4%)	43 (60.6%)	55 (51.9%)	0.27
Current	41 (23.2%)	12 (16.9%)	29 (27.4%)	
Former	38 (21.5%)	16 (22.5%)	22 (20.8%)	
Alcohol Use Disorder, no. (%)	86 (48.6%)	32 (45.1%)	54 (50.9%)	0.44
Illicit Drug Use, no. (%)	5 (2.9%)	1 (1.4%)	4 (3.8%)	0.65
Hand Dominance, no. (%)				
Right	121 (68.4%)	49 (69%)	72 (67.9%)	0.35
Left	8 (4.5%)	5 (7%)	3 (2.8%)	
Unknown	48 (27.1%)	17 (23.9%)	31 (29.2%)	N/A
Tear on MRI, no. (%)	169 (96.6%)	66 (94.3%)	103 (98.1%)	0.22
Severity of Tear, no. (%)				
Partial Thickness	41 (23.2%)	22 (31%)	19 (17.9%)	0.04
Full Thickness	136 (76.8%)	49 (69%)	87 (82.1%)	
Injury to Dominant Hand, no. (%)				
Non-dominant	54 (32.3%)	24 (35.8%)	30 (30%)	0.73
Dominant	78 (46.7%)	30 (44.8%)	48 (48%)	
Unknown	35 (21%)	13 (19.4%)	22 (22%)	N/A
Insurance Coverage				
Private	94 (54%)	36 (51.4%)	58 (55.8%)	0.13
Medicare	24 (13.8%)	14 (20%)	10 (9.6%)	
Medicaid	37 (21.3%)	14 (20%)	23 (22.1%)	
Other (Including Self, Military, Workers’ Comp)	12 (6.9%)	2 (2.9%)	10 (9.6%)	
Unknown	7 (4%)	4 (5.7%)	3 (2.9%)	
Workers’ Compensation, no. (%)	12 (6.8%)	1 (1.4%)	11 (10.5%)	0.03
Charlson Comorbidity Index, Median (IQR)	1 (1-2)	1 (1-2)	1 (0-1.5)	0.48
ASA Score, no. (%)				
1	23 (13.3%)	11 (15.9%)	12 (11.5%)	0.29
2	107 (61.9%)	45 (65.2%)	62 (59.6%)	
3	43 (24.9%)	13 (18.8%)	30 (28.8%)	
Myocardial Infarction, no. (%)	2 (1.1%)	1 (1.4%)	1 (1.0%)	1.0
Congestive Heart Failure, no. (%)	1 (0.6%)	0 (0%)	1 (1.0%)	1.0
Peripheral Vascular Disease, no. (%)	1 (0.6%)	1 (1.4%)	0 (0%)	0.40
Cardiovascular Disease, no. (%)	4 (2.9%)	2 (3.3%)	2 (2.6%)	1.0
Cerebrovascular Accident/Transient Ischemic Attack, no. (%)	3 (1.7%)	2 (2.8%)	1 (1.0%)	0.57
COPD, no. (%)	2 (1.1%)	0 (0%)	2 (1.9%)	0.52
Connective Tissue Disease, no. (%)	3 (1.7%)	0 (0%)	3 (2.9%)	0.27
Peptic Ulcer Disease, no. (%)	7 (4%)	3 (4.2%)	4 (3.8%)	1.0
Mild Liver Disease, no. (%)	15 (8.5%)	3 (4.2%)	12 (11.4%)	0.11
Diabetes Mellitus, no. (%)	30 (17%)	13 (18.3%)	17 (16.2%)	0.71
Chronic Kidney Disease, no. (%)	12 (6.8%)	4 (5.6%)	8 (7.6%)	0.76
Leukemia, no. (%)	8 (4.5%)	4 (5.6%)	4 (3.8%)	0.72
Metastatic Solid Tumor, no. (%)	3 (1.7%)	3 (4.2%)	0 (0%)	0.06
HIV/AIDS, no. (%)	1 (0.6%)	0 (0%)	1 (1.0%)	1.0
Thyroid Disease, no. (%)	18 (10.2%)	9 (12.7%)	9 (8.6%)	0.38
Hypertension, no. (%)	93 (52.8%)	38 (53.5%)	55 (52.4%)	0.88
Hyperlipidemia, no. (%)	79 (44.9%)	37 (52.1%)	42 (40%)	0.11

Univariate analyses for traumatic vs. atraumatic RCT injuries demonstrated that female patients were associated with decreased odds of traumatic RCT (female vs. male OR = 0.32, 95% CI: 0.17-0.60, p < 0.001) (Table 2). There were higher odds of having a full-thickness tear in the traumatic cohort (full vs. partial thickness OR 2.06, 95% CI: 1.01-4.16, p = 0.05) Patients insured by workers’ compensation were associated with greater odds of traumatic injury (workers’ compensation vs. none OR 8.19, 95% CI: 1.03-64.9, p = 0.05).

**Table 2:** Significant unadjusted and adjusted odds ratios for traumatic tear versus traumatic tear

Variable Name	Unadjusted Odds Ratio (95% CI) for Traumatic vs. Atraumatic	p-value	Adjusted Odds Ratio (95% CI) for Traumatic vs. Atraumatic	p-value
Sex				
Male	Reference	Reference	Reference	Reference
Female	0.32 (0.17-0.60)	<0.001	0.38 (0.20-0.74)	0.005
Severity of Tear				
Partial Thickness	Reference	Reference	Reference	Reference
Full Thickness	2.06 (1.01-4.17)	0.05	1.75 (0.83-3.67)	0.14
Workman’s Compensation				
No	Reference	Reference	Reference	Reference
Yes	8.19 (1.03-64.94)	0.05	5.51 (0.68-44.68)	0.11

In a multivariate logistic regression model (AIC = 227.119, BIC = 239.567, p<0.001, AUC = 0.67143) with sex, severity of tear, and workers’ compensation as factors, only sex remained significantly associated with traumatic RCT (female vs. male aOR 0.38, 95% CI: 0.20-0.74, p = 0.01, Table 2).

## Discussion

This study found that a higher proportion of patients under the age of 50 experienced a traumatic RCT. Additionally, traumatic tears were more likely to occur in males and were predominantly full-thickness tears. There were no other differences in demographic factors or comorbidities between the traumatic and atraumatic cohorts. While prior studies in patients less than 50 years of age with RCTs have reported demographics and clinical characteristics among their cohorts, there lacks a clear consensus on the impact of these variables on the etiology of tears in this patient population [7-11]. In consideration of the low prevalence of RCTs in young patients, the goal of this study was to broadly categorize all patients less than 50 years of age who sustained an RCT from our institution into cohorts based on etiology and then assess demographic factors and clinical characteristics associated with traumatic versus atraumatic RCTs. With limited research conducted into younger patients who have experienced atraumatic RCTs, we wanted to examine possible predisposing factors in this patient cohort.

Our retrospective analysis found that a greater number of young patients suffered a traumatic RCT when compared to the number of atraumatic tears. When patients were stratified into those two cohorts, the severity of the tear, sex, and workers’ compensation status were factors noted to be statistically significantly different. Comorbidities, including myocardial infarction (MI), congestive heart failure (CHF), peripheral vascular disease (PVD), cardiovascular disease (CVD), cerebrovascular accident/transient ischemic attack (CVA/TIA), chronic obstructive pulmonary disease (COPD), mild liver disease, diabetes mellitus (DM), chronic kidney disease (CKD), human immunodeficiency virus/acquired immunodeficiency syndrome (HIV/AIDS), thyroid disease, hypertension (HTN), and hyperlipidemia (HLD), were also evaluated by cohort and found to have no significant relationship. Previous studies evaluating predisposing factors for RCT found that those of traumatic origin were more commonly found in the younger population [8,22,23]. In their systematic review, Lazarides et al. reported that RCTs in young patients (age 16-40) are mainly traumatic in nature with a smaller proportion being due to elite throwing [8]. A more recent systematic review by MacKechnie et al. reported a prevalence of traumatic RCTs of 81% (60-100%) over seven studies examining patients under 55 years of age [12]. Moreover, these tears are found to be associated with greater size but decreased muscle atrophy and tendon retraction [14]. Additional research has demonstrated that RCTs in younger patients are more likely to be full-thickness regardless of etiology [7]. This corresponds to the trend demonstrated in our findings. Our study also demonstrated that traumatic tears were significantly more likely to be full-thickness and as well as associated with a work-related injury.

This study also found female patients have decreased odds of sustaining a traumatic RCT compared to male patients in both univariate and multivariate models. There have been numerous studies reporting varying results regarding the significance of sex as a risk factor for RCT [3,24-28]. Consistent with our findings, previous literature has reported that traumatic tears are more prevalent in men [24]. Additionally, Sambandam et al. demonstrated that males under 55 years of age were more likely to have larger tears than females of the same age group [2]. These findings also align with those found by Razmjou et al. in two separate studies demonstrating a significant association between sex and injury severity, which found women were more likely to have a smaller tear size and less severe injury compared to men [29,30]. More recently, Rai et al. found transcription level differences in the microbiology of the subacromial bursa in traumatic and degenerative tears that differed based on sex [31]. This may indicate that there may potentially be a sex-specific factor at the cellular level that predisposes young males to traumatic tears and females to degenerative tears. Future studies should focus on better understanding the sex-specific lifestyle factors that predispose males to increased odds of traumatic RCT.

Though our analysis did not identify any additional demographics or comorbidities that were significantly different among our cohorts, there are a few worth highlighting due to the conflicting literature currently available. Previous studies have shown an association between both a high BMI and HLD and the presence of rotator cuff disease [13,32]. Gumina et al. demonstrated that excess adiposity reflected through elevated BMI and percent body fat was associated with an increased risk of RCTs [13]. Additionally, in three-quarters of their cases of RCTs among cohorts, smoking or a medical condition predisposing to tear, such as HLD or thyroid disease, were present [11]. However, a major difference between our study and the one conducted by Gumina et al. was the average age of the patient. In the study by Gumina et al., the average age was 65.5 ± 8.52 years which differed significantly from our overall median age of 46 (IQR 42-48) [13]. Giri et al. also demonstrated an increased risk of RCT in patients who have HTN and HLD, which are typically comorbid conditions with each other as well as an elevated BMI [33]. While we did not find a difference in the rates of HTN and HLD between groups, more than 50% of the patients under the age of 50 with a tear did have HTN and more than 50% of our degenerative tears also had HLD. Additionally, when further evaluating the average age of the study populations included in the systematic review by Giri et al., there was only one study by Bodin et al. that had an average age younger than ours with females at a mean of 38.9 ± 10.3 and males at 38.5 ± 10.4 [34]. The other seven studies in the review consisted of cohorts with older patients than our own [33]. The average BMI in our cohort was also approaching obesity (29.9) but was not different between groups. Some of the other theorized potential risk factors for RCT include limb dominance [35] and smoking status [2,26,36]; however, these variables have shown variable significance within the literature [37-40]. Our study found that these did not prove to be risk factors for either tear type and there was no difference in distribution between the two cohorts. These findings suggest that there may be additional, age-specific predictive factors associated with traumatic versus atraumatic RCT that should be elucidated with future investigational studies.

A more recent study conducted by Guevara-Alvarez et al. examined the healing rates of traumatic and atraumatic tears in patient cohorts with average ages of 53 and 57 in the traumatic and atraumatic cohorts, respectively [41]. When examining their patient population's demographics, it was similar to ours, with no differences in HTN, HLD, or obesity and only showing a significant difference that atraumatic tears were more likely to be older and female. Many studies such as the one described by Guevara-Alvarez et al. evaluate the factors associated with RCT healing. However, fewer studies are examining predisposing factors that may lead to an RCT, specifically in younger patients. Although atraumatic tears are less prevalent in the younger population, they remain an area of interest for many as there continues to be difficulty in finding patient demographics that may predispose a young patient to an RCT.

The limitations of our study are consistent with the innate limitations of a retrospective study including an inferior level of evidence compared to prospective trials and a risk for sampling bias. Furthermore, our patient population was collected through the institutional electronic medical record via ICD-10 codes for RCT. Therefore, patients treated for RCTs at our institution may have been unintentionally excluded based on varying codes being utilized. Additionally, our study compared two cohorts composed of patients with RCT to identify potential risk factors for RCTs based on the etiology of the injury. We did not include a control group consisting of patients without RCTs and therefore are unable to definitively make conclusions about risk factors for our cohorts. However, the primary goal of this study was to contribute to the existing literature and help to further elucidate the differences in demographic and predisposing factors unique to the development of traumatic versus atraumatic RCT in patients younger than 50. The role of the demographics that were identified to be significantly different in our study may predispose young patients to RCT and may warrant further investigation.

## Conclusions

Patients under 50 years of age with an RCT are more likely to traumatic. Traumatic tears were more likely to be full thickness, occur in men, and result from a work injury. No other differences of statistical significance were noted in factors such as smoking status, limb dominance, or comorbidities such as hypertension and hyperlipidemia. Though we did not find any differences, additional studies may be beneficial to delve into the effect of medical comorbidities on the development of an RCT, specifically atraumatic, in this patient population. The factors that we did identify as different may be considered as possible targets for future, higher-powered studies.
